# Rearfoot and forefoot footfall patterns: implications for barefoot running

**DOI:** 10.1186/1757-1146-5-S1-K1

**Published:** 2012-04-10

**Authors:** Joseph Hamill

**Affiliations:** 1 University of Massachusetts, Amherst, MA, 01003, USA

## 

Approximately 75-80% of runners initiate contact with the running surface on their heel and thus have a rearfoot or heel-toe footfall pattern. The remaining 20-25% initially contact the ground with the foot flat with a subsequent heel contact (midfoot pattern) or on their forefoot with no heel contact (forefoot pattern). It is unclear why different footfall patterns exist or why some runners naturally use different patterns. Some contemporary training programs advocate the adoption of a mid- or forefoot footfall pattern but there is little scientific evidence that a particular strike pattern is more efficient or less injury-prone than other patterns. In this presentation, several studies that investigated differences among the footfall patterns relative to oxygen consumption, impact characteristics, surface alterations and lower extremity coordination will be presented. In addition, two modeling studies will also be discussed. One study will determine, using optimization techniques, the passive and active characteristics on the triceps surae. The other study is a forward dynamics study with different cost functions describing the different footfall patterns. Our basic premise in these studies is that different footfall patterns serve different functional roles in human running: a heel- or midfoot strike is used for endurance running, and a forefoot strike is used for sprinting. We propose that one’s footfall pattern is an intrinsic dynamic and thus difficult to alter. However, the change from shod to barefoot running often requires an alteration in footfall pattern that may ultimately lead to injury.

